# Fatty change of the liver microenvironment influences the metastatic potential of colorectal cancer

**DOI:** 10.1111/iep.12371

**Published:** 2020-08-11

**Authors:** Satoshi Masaki, Yoshimi Hashimoto, Shoma Kunisho, Akiko Kimoto, Yasuhiko Kitadai

**Affiliations:** ^1^ Department of Health and Science Prefectural University of Hiroshima Hiroshima Japan

**Keywords:** colorectal cancer, fatty liver, metastasis, organ microenvironment

## Abstract

Fatty liver is the most common cause of liver disease, and its prevalence has been increasing globally. Colorectal cancer (CRC) accounts for approximately 10% of all cancers and metastasizes most commonly to the liver. Paget's ‘Seed and Soil’ theory of metastasis proposed that the secondary growth of cancer cells is dependent on the distal organ microenvironment. This implies that the risk of metastasis may change due to changes in the microenvironment of target organs. However, the association between steatosis, fatty change in the liver microenvironment, and liver metastasis has not been clarified. Here, we induced fatty liver conditions in BALB/c mice using a choline‐deficient high‐fat diet with 0.1% methionine (CDAHFD) and then injected the CT26 cells to produce experimental metastasis. The number of metastatic tumours was significantly increased in mice with severe fatty liver as compared to control mice. The average size of metastatic tumours was smaller in mice with moderate fatty liver than in control mice. The stromal components, including cancer‐associated fibroblasts, tumour‐associated macrophages and tumour‐infiltrating lymphocytes, were also examined. Metastatic tumours in fatty liver showed invasive growth patterns without a fibrotic capsule. Compared to control groups, the polarization of macrophages and subtypes of tumour‐infiltrating lymphocytes differed depending on the extent of fatty liver progression. These results indicated that fatty changes in the liver influenced liver metastasis of CRC. Although moderate fatty changes suppress the growth of metastatic tumours in the liver, a severe fatty microenvironment may promote invasion and metastasis through alteration of the tumour microenvironment (TME).

## 1 INTRODUCTION

Fatty liver is characterized by the accumulation of triglycerides in hepatocytes and has been recently recognized as one of the most clinically important diseases because it can develop into cirrhosis and hepatic cancer. Non‐alcoholic fatty liver disease (NAFLD) is a disease that excludes alcoholic liver injury and other causes of secondary hepatic steatosis. It includes two distinct conditions with different histological characteristics and prognosis: non‐alcoholic fatty liver (NAFL) and non‐alcoholic steatohepatitis (NASH).^1^ The obesity epidemic has been closely linked with the rising prevalence of NAFLD,^2^ and the estimated prevalence of NAFLD is approximately 30‐40% in men and 15‐20% in women.^3^

Colorectal cancer (CRC) is the second most common cancer in men and the third most common cancer in women.^4^ There are 1.36 million people in the world who suffer from CRC, accounting for almost 10% of all cancers.^5^ The number of cases with good prognosis is increasing due to increased recognition and advances in diagnostic techniques, but there are still many advanced cancer cases. Colorectal cancer liver metastasis (CRCLM) is the leading cause of death for CRC.^6^ Thus, CRCLM is a significant problem to address to improve patient prognosis. Several risk factors for fatty liver and CRC, such as obesity and excessive fat intake, overlap,^7^, ^8^ and the number of CRC cases with fatty liver is expected to increase in developed countries.

The organ specificity of metastasis involves two factors: the blood circulation that carries cancer cells and the ability to selectively grow in specific organs. The ‘Seed and Soil’ theory of metastasis proposed by Paget in 1889 suggested that the metastasized organ microenvironment is suitable for the engraftment and proliferation of cancer cells.^9^ The organ that colon cancer is most likely to metastasize to is the liver,^5^ and this is not only due to an anatomical factor, but also because the liver has a microenvironment in which colon tumour cells can easily grow. Tumour cells interact with stromal cells such as fibroblasts, macrophages, and vascular constituent cells through soluble mediators such as growth factors and cytokines, and tumour‐stromal cell interactions have an important role in the process of metastatic foci formation.^10^ Colonization is the most difficult step among the multiple steps of metastasis, and only 0.02% of circulating cancer cells can form metastatic tumours.^11^, ^12^ Therefore, fatty changes in the liver microenvironment may influence the metastatic potential of CRC.

In the present study, we examined how fatty changes of the liver influence liver metastasis using a choline‐deficient high‐fat diet with 0.1% methionine (CDAHFD) for mice. Histological analysis was also performed to elucidate the role of stromal cells in the tumour microenvironment.

## 2 MATERIALS AND METHODS

### 2.1 Animals and diet study

Five‐week‐old female BALB/c mice (Charles River Japan) were used. Fatty liver was induced by feeding a choline‐deficient high‐fat diet with 0.1% methionine (CDAHFD: #A06071302, Research diet) for up to 6 weeks, and the mice in the control group received a control diet (CD:MF, Oriental Yeast). Food and liquids were given ad libitum and exchanged twice a week, and the mice were weighed weekly. Fatty liver was evaluated every 2 weeks (n = 3). Body and liver weights were measured on sacrifice, and the obtained liver tissue was fixed with zinc and embedded in paraffin, and used for histological evaluation. The liver tissues were stained with haematoxylin and eosin (HE) to assess the NAFLD Activity Score (NAS), following previous protocols.^13^ Fibrosis was evaluated by Masson's trichrome staining.

### 2.2 Ethical approval

All experimental procedures were performed in compliance with the relevant laws and institutional guidelines and have been approved by the local ethics committees.

### 2.3 Tumour cell line

CT26 cells were used as a syngeneic mouse colon cancer cell line. Cells were maintained in Dulbecco's modified Eagle's medium (DMEM) with 10% foetal bovine serum (FBS) and a penicillin‐streptomycin mixture at 37℃ and 5% CO₂. Prior to transplantation, cell suspensions were adjusted to the proper concentration using a TC20 automated cell counter (Bio‐Rad, Richmond, CA) with trypan blue dye (0.40%) and suspended in calcium‐ and magnesium‐free Hank's balanced salt solution.

### 2.4 In vivo tumour transplantation

Our preliminary study has shown that moderate steatosis can be induced by feeding CDAHFD for 2 weeks, and severe steatosis can be induced by feeding CDAHFD for 4 weeks. Therefore, mice were divided into the following 4 groups: control (2w) group (2‐week CD feeding before transplantation), moderate fatty liver group (2‐week CDAHFD feeding before transplantation), control (4w) group (4‐week CD feeding before transplantation) and severe fatty liver group (4‐week CDAHFD feeding before transplantation) (n = 9, 10, 10 and 11/group respectively). After feeding CD or CDAHFD, a left subcostal surgical incision was created and 1 × 10^5^ tumour cells/50 μL was injected into the exposed spleen of each mouse. Fourteen to fifteen days after transplantation, the animals were sacrificed and liver weight, number of macrometastasis and size of micrometastasis were measured.

The liver tissue, including the tumour, was collected, zinc‐fixed, paraffin‐embedded and used for histological examination. In addition, the tumour tissue was collected and immediately stored at −80℃ and used for isolation of total RNA in quantitative real‐time PCR (qPCR).

### 2.5 Immunohistochemistry and Immunofluorescence analysis

At sacrifice, the livers containing tumours of each mouse were sampled, fixed in zinc‐formalin and sliced to 4‐µm sections. Immunohistochemistry was performed using the Envision^TM^ + system (DAKO), and immunofluorescence staining was performed using the Opal Fluorescent IHC Kit (PerkinElmer) according to the manufacturer's instructions. The MI‐77 MW rapid processor (Azumaya) was used for some reactions in the process. Immunohistochemistry was performed using the following antibodies: anti‐Ki‐67 (GTX16667, GeneTex); anti‐CD31 (ab28364, Abcam); anti‐αSMA (ab5694, Abcam); anti‐FAP (ab53066, Abcam); anti‐CD4 (#25229, Cell Signaling); anti‐CD8α (#98941, Cell Signaling); and anti‐FOXP3 (#12653, Cell Signaling). Immunofluorescence staining was performed utilizing the following antibodies: anti‐F4/80 (ab6640, Abcam); anti‐NOS2 (sc‐651, Santa Cruz); anti‐CD163 (ab182422, Abcam); and anti‐VEGF‐A (sc‐152, Santa Cruz). A confocal microscope (FV1000D, Olympus) was used for fluorescence observation. For all the studies, randomly selected fields (100 × magnification) for every large metastasis (ie those> 1 mm^2^) were analysed using WinROOF version 3.6 (Mitani).

### 2.6 RNA isolation, cDNA synthesis and qPCR

During tissue collection, a tumour from each animal was rapidly frozen and used for RNA isolation. Total RNA was isolated with the NucleoSpin TriPrep Kit (Takara Bio Inc., Shiga, Japan) according to the manufacturer's protocol. After confirming the purity and concentration of RNA, 1000 ng of total RNA from each sample was reverse‐transcribed to cDNA using the QuantiTect Reverse Transcription Kit (Qiagen) according to the manufacturer's protocol. qPCR reactions with cDNA were performed using the QuantiTect SYBR® Green PCR Kit (Qiagen). The LightCycler (Roche Diagnostics) was used for the reaction, normalized by the Pfaffl analysis method [23]. GAPDH served as the internal control. The primer sequences were as follows: IFN‐γ, forward primer 5′‐GGATGCATTCATGAGTATTGC‐3′ and reverse primer 5′‐GTGGACCACTCGGATGAG −3′; TNF‐α, forward primer 5′‐CCACCACGCTCTTCTGTCTAC‐3′ and reverse primer 5′‐AGGGTCTGGGCCATAGAACT‐3′; IL‐12p40, forward primer 5′‐CTTGTTCGAATCCAGCGCA‐3′ and reverse primer 5′‐TAGCGATCCTGAGCTTGCAC‐3′; TGF‐β, forward primer 5′‐TTGCTTCAGCTCCACAGAGA‐3′ and reverse primer 5′‐TGGTTGTAGAGGGCAAGGAC‐3′; IL‐10, forward primer 5′‐CAAGGAGCATTTGAATTCCC‐3′ and reverse primer 5′‐GGCCTTGTAGACACCTTGGTC‐3′; perforin, forward primer 5′‐AACCTCCACTCCACCTTGAC‐3′ and reverse primer 5′‐GTGCGTGCCATAGGAGGAGA‐3′; FASL, forward primer 5′‐GCAGCAGCCCATGAATTACC‐3′ and reverse primer 5′‐AGATGAAGTGGCACTGCTGTCTAC‐3′; and GAPDH, forward primer 5′‐GGGTGAGGCCGGTGCTGAGTATG‐3′ and reverse primer 5′‐GGCAGAAGGGGCGGAGATGATG‐3′.

### 2.7 Statistical analysis

All data were given as mean ± SEM. Statistical comparisons between two groups were performed using t tests following evaluation of equality of variance or not with Levene's test. Significance level was set at *P* < .05. All statistical testing was performed using SPSS Statistics 23.0 (IBM Japan).

## 3 RESULTS

### 3.1 The histopathological evaluation of CDAHFD‐induced fatty changes of the liver

**Figure 1:**
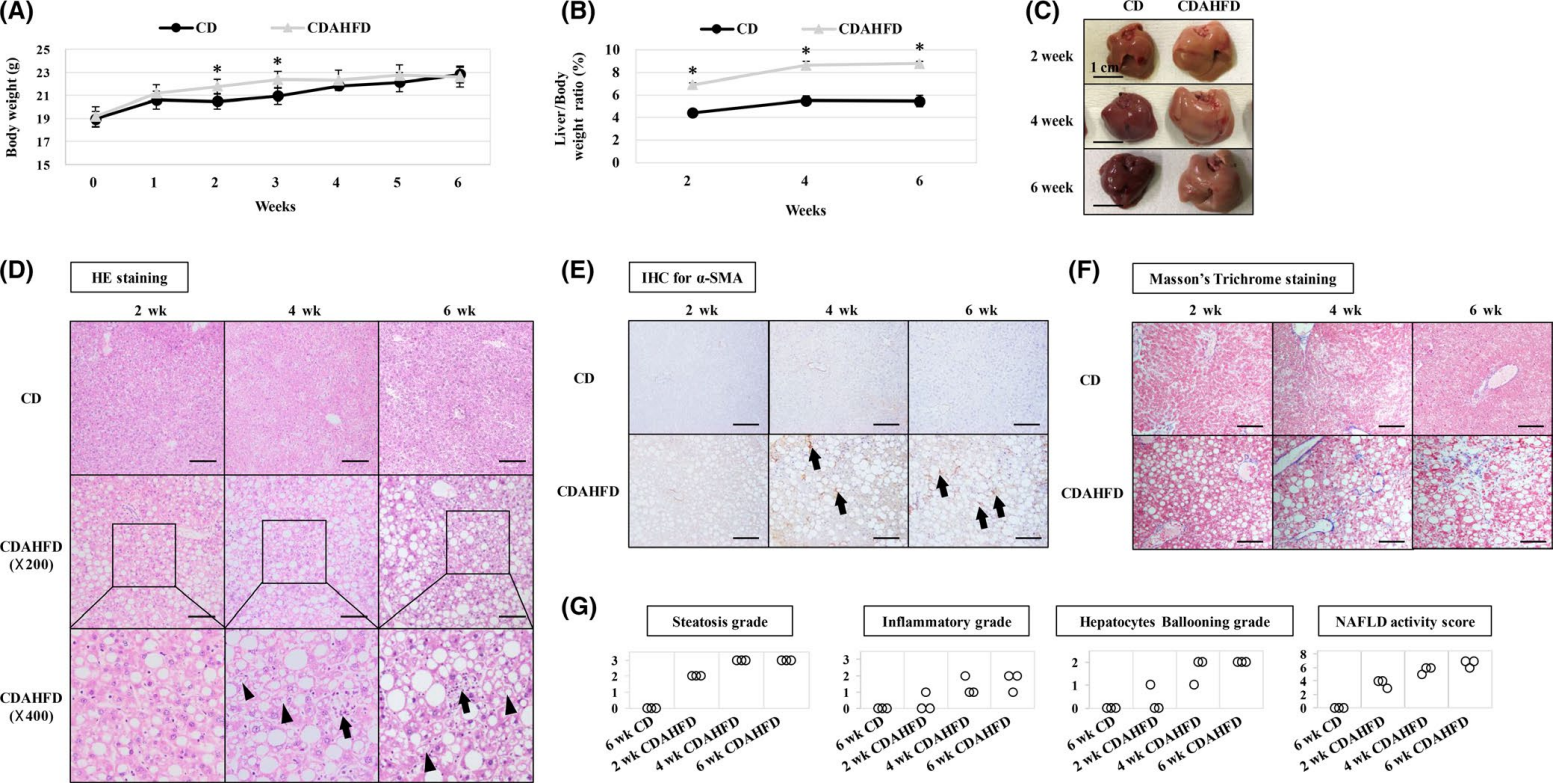
Histopathological evaluation of CDAHFD‐induced fatty liver. A) Body weight change in each group. The CDAHFD group showed an increase in body weight as well as the control group. B) Change in liver/body weight ratio. The CDAHFD group increased in liver/body weight ratio. C) Macroscopic findings of the liver. In the CDAHFD group, liver hypertrophy and whitening are observed. D) HE staining of liver tissue. In the CDAHFD group, accumulation of lipid droplets was observed after 2 wk. Hepatocyte ballooning (arrow) and inflammatory cell infiltration (arrowhead) appeared and increased at each time period. E) Representative images of immunohistochemistry (IHC) for α‐SMA. In the CDAHFD group, activation of hepatic stellate cells that was not observed after 2 wk was observed after 4 wk (arrow). F) Representative image of Masson's trichrome staining. After 6 wk of giving CDAHFD, very mild portal fibrosis was observed. G) Evaluation of disease severity by NAFLD activity score (NAS). Five points or more of NAS was diagnosed as NASH. After 4 wk, the condition was mainly equivalent to NASH. *P < .05, **P < .01, scale bars represent 100 μm unless otherwise indicated

To evaluate whether CDAHFD intake actually induces fatty change in the liver, CD or CDAHFD was given for up to 6 weeks, and body and liver weight were measured. The CDAHFD groups showed an increased trend in body weight similar to the CD groups (Figure 1A). The liver/body weight ratio increased significantly from 2 weeks after feeding CDAHFD (Figure 1B), and hepatic hypertrophy and whitening were observed macroscopically (Figure 1C). Histopathological evaluation by HE staining revealed accumulation of lipid droplets in hepatocytes and low inflammatory cell infiltration after 2 weeks, but almost no hepatocyte ballooning was observed. Steatohepatitis such as infiltration of inflammatory cells and ballooning was observed after 4 weeks (Figure 1D). Masson's trichrome staining revealed mild portal fibrosis after 6 weeks (Figure 1E). In addition, activated hepatic stellate cells were observed after 4 weeks (Figure 1F). Hence, the progression of fatty liver in this model was similar to that of humans. According to the histopathological diagnosis by NAS, moderate fatty liver (NAS 3 or 4) became clear after 2 weeks, and severe fatty liver (NAS> 5) became clear after 4 weeks (Figure 1G).

### 3.2 Influences of fatty change in organ microenvironment on liver metastasis

**Figure 2:**
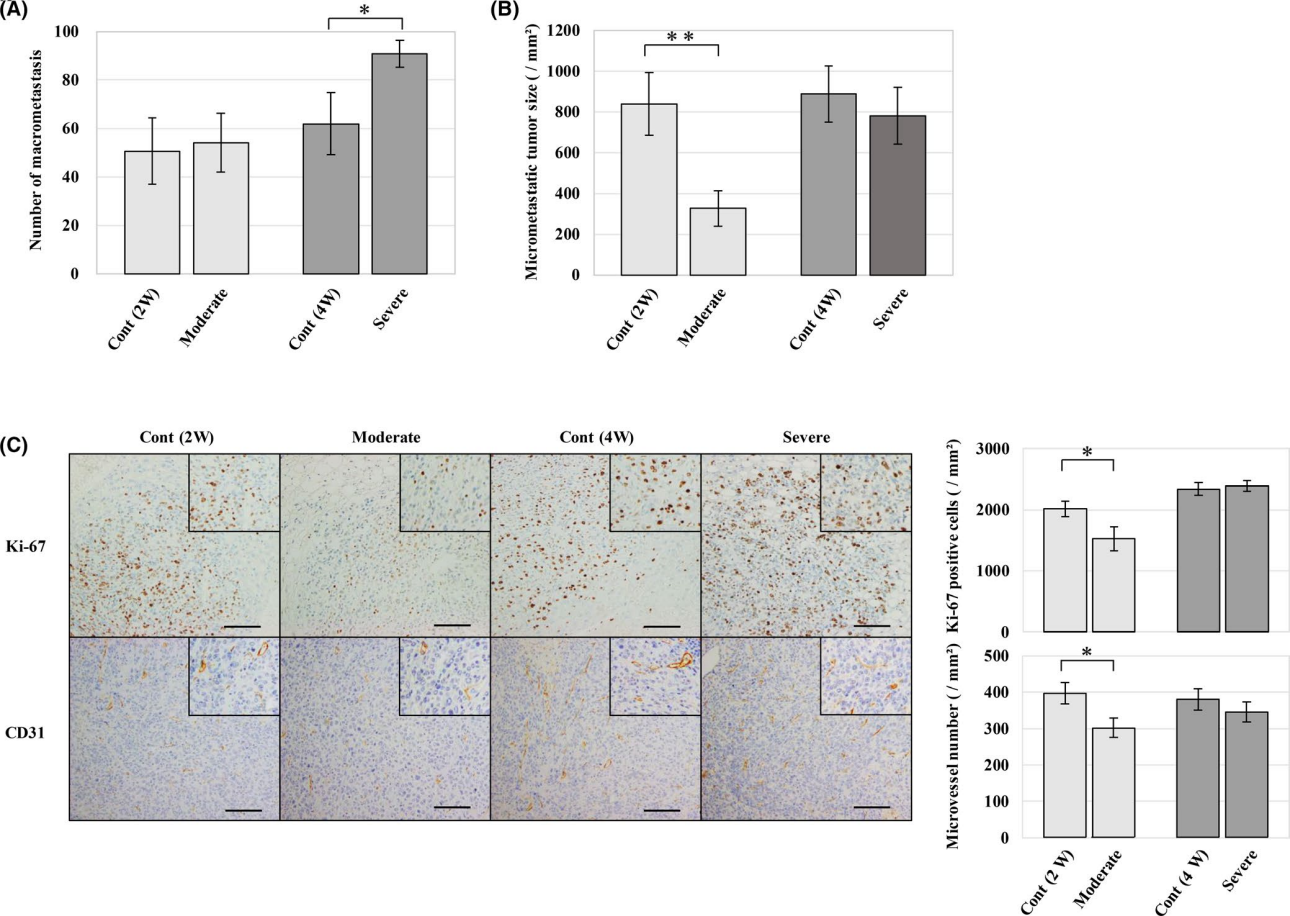
Effects of fatty change in liver microenvironment on liver metastasis. A) Comparison of the number of metastases. The number of metastases did not change in the moderate fatty liver group but increased in the severe fatty liver group. B) Evaluation of micrometastatic tumour size. There were smaller metastatic lesions in the moderate fatty liver group. C) Representative images of immunohistochemistry (IHC) for Ki‐67 and CD31, and analysis of positive cells in metastatic tumour. Compared to the control group, the moderate fatty liver group showed a significant decrease in Ki‐67‐positive cells and microvessel number. *P < .05, **P < .01, scale bar represents 100 μm

To clarify how fatty liver development affects liver metastasis of colon cancer cells, CT26 cells were experimentally injected into the liver of mice fed CD or CDAHFD for 2 or 4 weeks. In this study, we defined fatty liver after 2 weeks as moderate and 4 weeks as severe. As a result, the tumour was engrafted at the primary site and metastasis occurred in most mice, while there was no difference between the groups (data not shown). The number of metastatic tumours was significantly higher in the severe fatty liver group as compared to the corresponding control group. The size of metastatic tumours was smaller in the moderate fatty liver group (Figure 2B). There was no difference in spleen weight and primary tumour volume (data not shown).

In addition, we performed immunohistochemistry using anti‐Ki‐67 and anti‐CD31 antibodies to evaluate the proliferation and angiogenesis of metastatic tumours. As a result, compared with each control group, the moderate group had significantly fewer Ki‐67‐positive cells in the metastatic tumour (Figure 2C). Similarly, evaluation of microvessel number also revealed that there were fewer microvessels in the moderate group (Figure 2C).

### 3.3 Histopathological evaluation of fatty change in the tumour microenvironment

Tumour‐stromal cell interactions in the TME play an important role in tumour metastasis. So, we next examined whether fatty changes of the liver affect CRCLM by modulating the TME.

**Figure 3:**
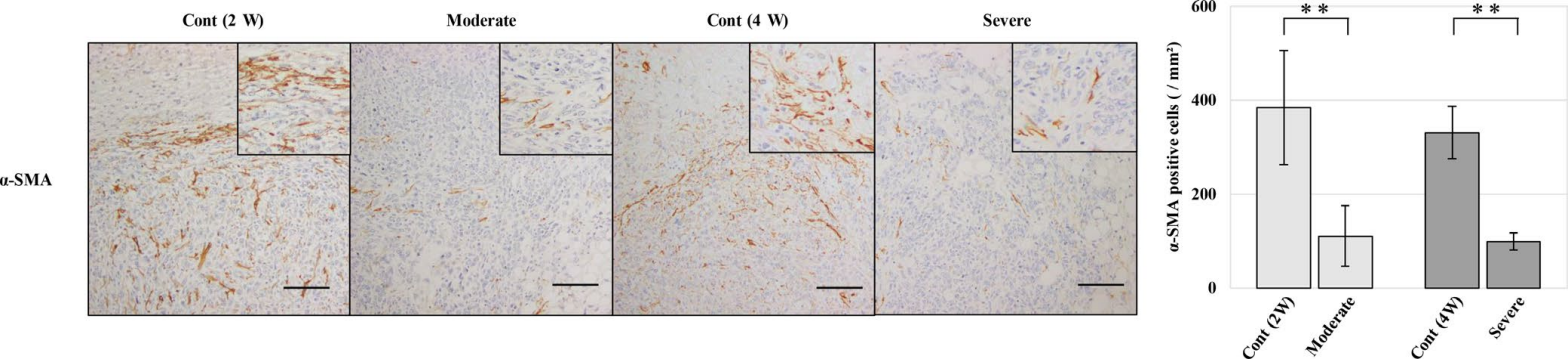
Immunohistochemistry for different markers of cancer‐associated fibroblasts (CAFs) in a metastatic tumour. Representative images of α‐SMA staining and analysis of positive cells in a metastatic tumour. Each fatty liver group reduced α‐SMA‐positive cells in metastatic tumours. ** *P* < 0.01, scale bar represents 100 μm

Cancer‐associated fibroblasts (CAFs) are the principal component of stromal cells within the TME and contribute to tumour progression. CAFs in tumours were evaluated by immunohistochemistry for α‐SMA and FAP. In the control group, an accumulation of α‐SMA‐positive cells was observed at the tumour periphery. Tumours showed expanding growth patterns encapsulated with CAFs. However, metastatic tumours in both the moderate and severe fatty liver groups showed invasive growth without fibroblastic encapsulation by α‐SMA‐positive cells at the tumour periphery. There were significantly few α‐SMA‐positive cells in the metastatic tumour compared to each control group (Figure 3). FAP‐positive cells also showed the same tendency as α‐SMA; FAP expression was not significantly different between the groups (data not shown).

**Figure 4:**
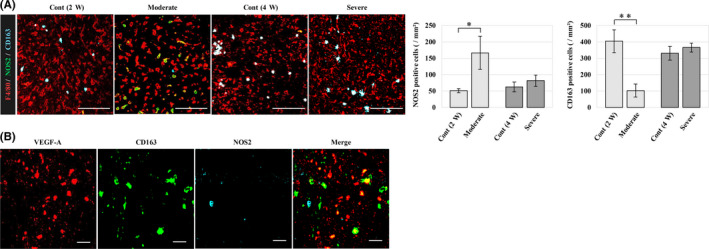
Phenotype classification of macrophages in the metastatic tumour microenvironment. A) Immunofluorescence with total macrophage, M1 and M2 macrophage markers (red, F4/80; green, NOS2; cyan, CD163). Compared with the other groups, the moderate fatty liver group had more M1 macrophages in the tumour, and conversely, there were fewer M2 macrophages, and differences of polarization were not seen in the severe fatty liver group. Scale bar represents 100 μm. B) Fluorescent staining with VEGF‐A (Red) and each macrophage marker (CD163, cyan; NOS2, green). Scale bar represents 20 μm. M2 macrophages expressed VEGF‐A, which was not expressed by M1 macrophages. * *P < .05*, ** *P < .01*

Next, the polarization of tumour‐associated macrophages (TAMs) was evaluated by immunofluorescence. F4/80, NOS2 and CD163 were used as markers for total macrophages, M1 macrophages and M2 macrophages respectively. In the moderate fatty liver group, a large number of M1 macrophages were observed in the tumour compared to the control group, and conversely, a small number of M2 macrophages were observed as compared to the control group (Figure 4A). In contrast, such differences were not observed in the severe fatty liver group, and the subtype of TAMs in the metastatic tumour was different depending on the development of fatty liver. Furthermore, we evaluated the expression of VEGF‐A in M1 and M2 macrophages where M2 macrophages expressed VEGF‐A (Figure 4B).

**Figure 5:**
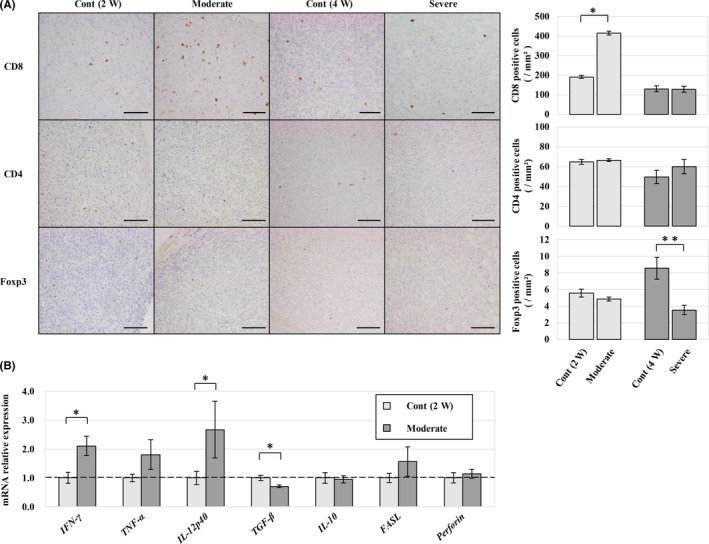
Early fatty liver enhances anti‐metastatic tumour immunity. A) Immunohistochemistry of tumour‐infiltrating T cells in each group. There were almost no CD4‐positive or Foxp3‐positive cells in all groups. Numerous CD8 + cells were observed in the tumour, especially in the moderate fatty liver group. B) RNA expression level of immune‐related genes in moderate fatty liver group by qPCR using total RNA extracted from tumour tissue. A significant increase in expression was observed with IFN‐γ and IL‐12p40, and a significant decrease was observed with TGF‐β.*P < .05, ** *< .01*

Finally, changes in anti‐tumour immunity caused by fatty liver were evaluated. Intratumoral infiltration of T lymphocytes was evaluated by immunohistochemistry using CD4, CD8 and Foxp3 antibodies as markers for helper T, killer T and regulatory T cells respectively. CD4‐positive cells sparsely existed in the tumour, but there was no difference between the groups (Figure 5A). Tumour‐infiltrating CD8 + cells were significantly increased in the moderate fatty liver group (Figure 5A). Foxp3‐positive cells were rarely found and were significantly decreased in the severe fatty liver group (Figure 5A). Relative quantification of mRNA expression by qPCR was performed between the moderate fatty liver and control groups, which showed differences in tumour immune responses. Th1 cytokines, such as IFN‐γ and IL‐12p40, were significantly increased in the moderate fatty liver group, whereas Th2 cytokines, such as TGF‐β, were significantly decreased (Figure 5B).

## 4 DISCUSSION

Establishing metastasis requires the support of host stromal cells in the secondary organ, which means that the metastasis is influenced by the microenvironment of the secondary organ.^10^ Therefore, changes in the liver microenvironment caused by a fatty liver may affect liver metastasis of colon cancer cells.

However, the association between CRCLM and fatty liver is not yet clear. Bin Cai et al. concluded in a meta‐analysis that CRCLM is unlikely to occur in patients with chronic liver injury, including fatty liver, and suspected that the reason for this is not due to the primary tumour, but due to changes in the liver microenvironment.^14^ However, previous studies in mice have shown that fatty liver promotes liver metastasis in colorectal cancer.^15^, ^16^, ^17^ Hence, in this study, we examined how fatty changes in the liver microenvironment can affect CRCLM, focusing on the metastatic TME.

The fatty livers induced in this study did not lose weight. In addition, the pathological features of human fatty liver disease, such as hepatocyte ballooning and hepatic fibrosis with disease progression, were found to be reflective in the human fatty liver disease model. As a result of transplantation, metastatic tumour growth was suppressed in moderate fatty livers, and the number of metastases increased in severe fatty livers. These results suggest that moderate fatty liver suppresses tumour growth, and the microenvironment changes to a favourable microenvironment for tumour metastasis as fatty liver disease progresses. Anti‐Ki‐67 staining showed that metastatic tumour proliferation was suppressed in the moderate fatty liver group.

Hepatic stellate cells (HSCs) are activated and differentiated into a myofibroblast‐like phenotype with the development of fatty liver. Therefore, resident CAFs are expected to increase in the fatty liver. However, rather, metastatic tumours formed in fatty livers did not contain many CAFs. So far, it has been reported that metastasis of lymphoma cells to the fatty liver, induced by a choline‐deficient diet, is suppressed, and this is associated with a decrease in CAFs.^18^

TAMs are conventionally classified into the cancer‐inhibiting M1 types and cancer‐promoting M2 types.^19^ As a result of classification of TAMs in this study, there were many M1 types and few M2 types in the metastatic tumours in the moderate fatty liver. Furthermore, M2 types expressed VEGF‐A, and inhibition of differentiation to the M2 type contributed to suppression of metastatic tumour progression. However, this phenomenon did not occur in the severe fatty liver. Macrophage differentiation is regulated by complex interactions affected by the local microenvironment,^20^ and a severe fatty liver may trigger differentiation into tissue remodelling/profibrosis. In addition, the HSC activation revealed by anti‐α‐SMA staining, which was rarely seen in the moderate fatty liver, increased in the severe fatty liver. Activation of HSCs promotes the growth of cancer cells by inducing monocytes from M1 to M2 type.^21^, ^22^ Kupffer cells also play an important role in each step of liver metastasis formation. However, the role of Kupffer cells in metastasis is controversial.^23^, ^24^, ^25^

Tumour‐infiltrating lymphocytes (TILs) such as cytotoxic T cells are present locally in solid cancers and represent the anti‐tumour response of the host. Gooden et al. reported in a meta‐analysis that CD8 + cell infiltration is a good prognostic factor.^26^ In this study, many CD8 + T cells were found in the tumours of the moderate fatty liver group, suggesting that activation of tumour immunity may contribute to tumour elimination. Changes in mRNA expression levels of cytokines such as IFN‐γ and TGF‐β also suggest this possibility. These mechanisms include activation of the immune system in fatty liver^27^ and remodelling of the immunosuppressive tumour microenvironment through the interaction of stromal cells such as TAMs. Tumour‐infiltrating CD8 T cells were not increased in the severe fatty liver, and this is probably because M2 macrophages were the dominant cell type. Considering these findings, it is speculated that the CRCLM and metastatic proliferation are influenced due to the interaction between tumour cells and hepatic microenvironment changed with development of fatty liver, such as chronic inflammatory response and extracellular matrix remodelling.

Our findings that moderate fatty liver suppresses metastatic growth of colorectal cancer differ from previous studies.^15^, ^16^, ^17^ The difference may be due to the use of different study animals, cell lines or fatty liver models. For example, Tamura et al. showed that using F344 rat showed that the fatty liver is an unfavourable environment for liver metastasis of colon cancer.^28^ In chronic liver disease, macrophages and lymphocytes have different effects depending on the pathogenesis and stage of fatty liver, and very complicated mechanisms exist. In this study, NK cells and NKT cells have not been examined, but NKT cells are more abundant in the liver than in other organs. NKT cells are expected to be involved in this mechanism because they recognize glycolipid antigens and are closely associated with chronic liver disease.^29^, ^30^

In conclusion, the influence of fatty liver on CRCLM differs depending on the disease progression. The moderate fatty liver suppresses tumour growth, while the severe fatty liver becomes a favourable environment for tumour metastasis. One reason for this is a change in the microenvironment of metastatic tumours, which involves cancer‐stromal cell interactions.

## CONFLICT OF INTEREST

The authors declare that there is no conflict of interest regarding the publication of this article.
